# Contextual Hub Analysis Tool (CHAT): A Cytoscape app for identifying contextually relevant hubs in biological networks

**DOI:** 10.12688/f1000research.9118.2

**Published:** 2016-08-30

**Authors:** Tanja Muetze, Ivan H. Goenawan, Heather L. Wiencko, Manuel Bernal-Llinares, Kenneth Bryan, David J. Lynn

**Affiliations:** 1 EMBL Australia Biomedical Informatics Group, Infection & Immunity Theme, South Australian Medical and Health Research Institute, Adelaide, Australia; 2 Animal and Bioscience Research Department, Animal and Grassland Research and Innovation Centre, Teagasc, Meath, Ireland; 3 School of Medicine, Flinders University, Bedford Park, Australia

**Keywords:** Network analysis, hypergeometric test, hubs, gene expression data, contextual hub analysis, CHAT

## Abstract

Highly connected nodes (hubs) in biological networks are topologically important to the structure of the network and have also been shown to be preferentially associated with a range of phenotypes of interest. The relative importance of a hub node, however, can change depending on the biological context. Here, we report a Cytoscape app, the Contextual Hub Analysis Tool (CHAT), which enables users to easily construct and visualize a network of interactions from a gene or protein list of interest, integrate contextual information, such as gene expression or mass spectrometry data, and identify hub nodes that are more highly connected to contextual nodes (e.g. genes or proteins that are differentially expressed) than expected by chance. In a case study, we use CHAT to construct a network of genes that are differentially expressed in Dengue fever, a viral infection. CHAT was used to identify and compare contextual and degree-based hubs in this network. The top 20 degree-based hubs were enriched in pathways related to the cell cycle and cancer, which is likely due to the fact that proteins involved in these processes tend to be highly connected in general. In comparison, the top 20 contextual hubs were enriched in pathways commonly observed in a viral infection including pathways related to the immune response to viral infection. This analysis shows that such *contextual hubs *are considerably more biologically relevant than degree-based hubs and that analyses which rely on the identification of hubs solely based on their connectivity may be biased towards nodes that are highly connected in general rather than in the specific context of interest.

Availability: CHAT is available for Cytoscape 3.0+ and can be installed via the Cytoscape App Store ( http://apps.cytoscape.org/apps/chat).

## Introduction

Network analysis has emerged as a powerful approach to elucidate biological and disease processes ^1^. Biological networks (and many other types of networks) have been shown to have a power law distribution of node connectivity, with most nodes having few connections and a few nodes being highly connected ^2^. The identification of such highly connected nodes, termed hubs, is often of interest as hubs have been shown to be topologically and functionally important. The deletion of genes encoding hub proteins, for example, has been shown to correlate with lethality in yeast (the centrality-lethality rule) ^3^. Hubs have also been found to be preferentially targeted by both bacterial and viral pathogens ^4^ and may be master regulators of biological processes ^5^. Biological networks, such as the human interactome, however, are not static entities ^6^, and the extent to which a node acts as a hub can change depending on the biological context e.g. the network present in a specific cell type at a particular point in time ^7, 8^. Integrating contextual information, such as gene or protein expression data, with standard network analysis can provide insight into what are the most relevant network features in a particular study or context ^9– 11^.

Cytoscape has a number of applications to identify hubs in networks including cytoHubba ^12^, APID2Net ^13^, PinnacleZ ^14^, NetworkAnalyzer ^15, 16^ and CentiScaPe ^17^, however, only the latter two are compatible with Cytoscape 3+. All of the applications available to date identify hubs based on node connectivity (degree) in a network of interest. To construct a network, users frequently query interaction databases to identify the interactors of a list of genes of interest, e.g. differentially expressed genes, and then identify the high degree nodes in this network. This approach to constructing a network is useful because it identifies a more fully connected network for analysis than would be the case if one restricted interactions to only those that occur between nodes in the gene list. Analysis of these networks can, for example, identify subnetworks that are enriched in (but do not exclusively consist of) differentially expressed genes, or identify non-differentially expressed nodes that are topologically important in the network, both of which would otherwise not be identified. Identifying hubs in these networks, however, is biased towards identifying nodes that are highly connected in general such as promiscuous, ubiquitous or well-studied nodes, because nodes with many interactions in the query database have a higher probability of being included in the network by chance alone. Analysis of these degree-based hubs, for example identifying what biological processes or pathways these nodes are enriched in, tells us little about the experimental context of interest and more about the properties of highly connected nodes in general. A more appropriate analysis is to determine which nodes interact with relevant nodes in the network (which we term contextual nodes) more than is statistically expected.

Here, we introduce the Contextual Hub Analysis Tool (CHAT), a Cytoscape App that identifies hub nodes that interact with more "contextual" nodes (e.g. differentially expressed genes or proteins) than statistically expected in networks integrated with user-supplied contextual data (e.g. gene expression data). We term these nodes *contextual hubs*. We show that such *contextual hubs* are considerably more relevant than degree-based hubs to the specific experimental context under investigation. As such, these nodes are promising candidates for further functional validation studies and potentially represent important points in the network for drug targeting.

## Methods

### Implementation

CHAT was written in Java 8 as an Open Services Gateway Initiative (OSGi) bundle for Cytoscape 3.0+ ^18^. It adds a “CHAT” option in the “Apps” menu that launches a popup window, which allows users to adjust different network initialization parameters. CHAT prompts users to input a list of gene identifiers (the supported ID types are dependent on the database selected by the user) and any associated contextual data, e.g. gene expression data associated with the genes. While the focus of this paper is on genes, CHAT can equally be applied to proteins. The OK button triggers Cytoscape’s TaskManager to run a task that initiates the network construction and adds a tab to the results panel that provides functionality to further modify and analyze the network. To create the network, CHAT finds all the first neighbor interactors of the user-provided genes (or their encoded products). Interaction data is retrieved from one of the databases included in the PSICQUIC registry ^19^, which the user can select. Note that interactions between the first neighbors are considered by CHAT but these are not included in the network visualization for clarity reasons. Once the network has been constructed, CHAT performs a hypergeometric test on each node in the network to identify nodes that interact with contextual nodes more than expected by chance. The probability that a given hub has *k* or more contextual interactors among its *n* interactors is given by the hypergeometric distribution:

$p(X\geq k)=\sum_{x=k}^{n}\frac{\left(\begin{array}{l} K\\ x \end{array}\right)\left(\begin{array}{c} N-K\\ n-x \end{array}\right)}{\left(\begin{array}{c} N\\ n \end{array}\right)}$

Where *N* is the number of genes with at least one interaction in the database queried and *K* is the number of contextually relevant nodes provided by the user (with at least one interaction in the database queried). Overrepresentation analysis heavily depends on the choice of background dataset for the determination of *N*. To estimate the background frequency *K*/ *N*, CHAT provides access to interaction data from databases available in the PSICQUIC registry. Databases with less than 10,000 interactions are excluded. The number of genes in the user-selected database that have at least one interaction (of the specified type) in which both interactors match the user-selected criteria for constructing the network (species, interaction type and ID type) determine the node population size *N*. Self-interactions are disregarded. Interactions between input genes and between their first neighbors are considered in the CHAT analysis. P-values calculated by CHAT are automatically corrected for multiple testing using the Benjamini-Hochberg procedure ^20^, a method widely used in bioinformatics to avoid high false discovery rates. The Bonferroni approach is widely considered to be too strict ^21^.

A right click on a node brings up an option to activate the “Node Analyzer” mode, which allows the user to analyze the connectivity pattern of individual hubs of interest. Using this function will display the node analyzer table on the results panel and all nodes except the selected node and its interactors will be hidden in the network visualization. The execution time of CHAT varies between a few seconds and a few minutes based on the number of user-supplied (contextual) genes, the size of the chosen database and its connection speed as well as the user-selected network layout. These factors also influence memory consumption.

### Operation

**Figure 1.**
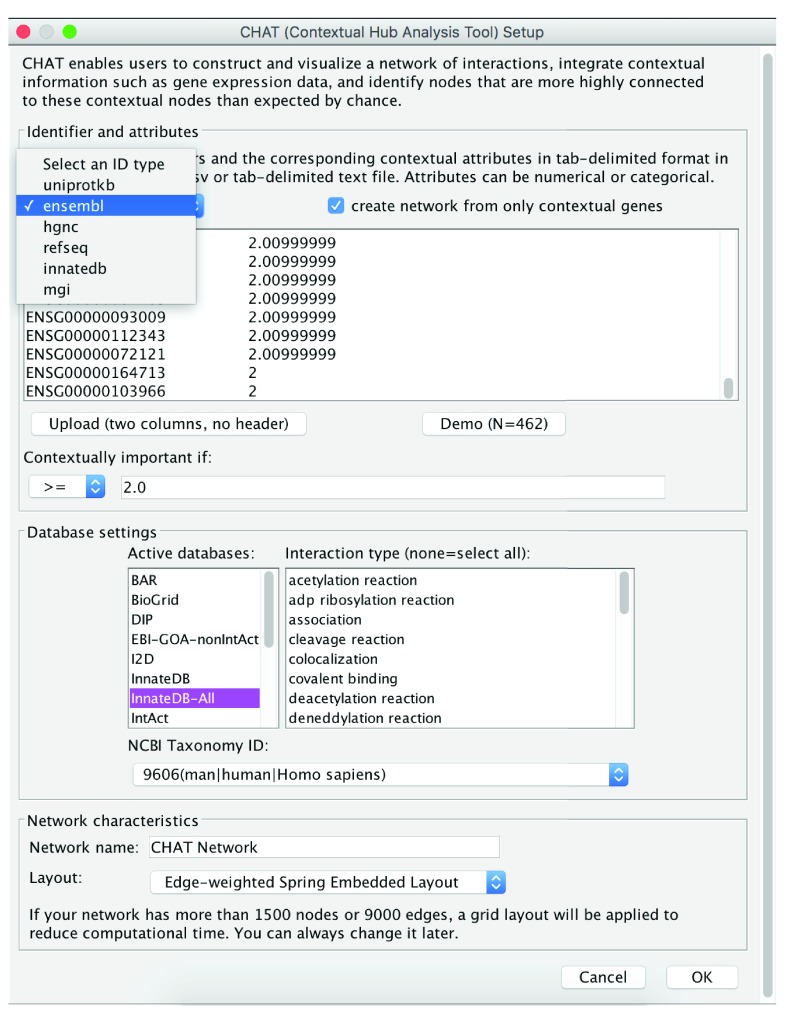
CHAT network analysis. To construct a network using CHAT, the user provides a list of gene identifiers and associated numerical or categorical attributes relevant in the context of interest.

**Figure 2.**
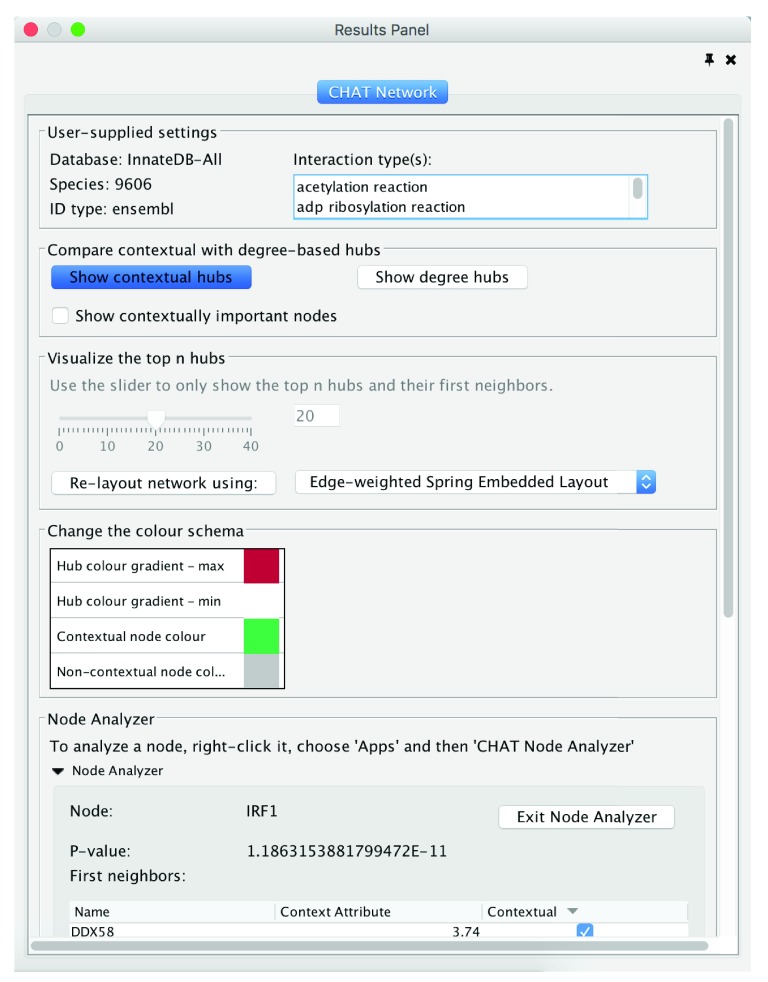
Network visualization. CHAT provides a number of options to customize the network visualization.

The identification of the top contextual hubs consists of three primary steps: 1) input of a user-supplied gene list and contextual data, 2) network construction and statistical analysis to identify nodes that preferentially interact with contextual nodes and 3) visualization of the top contextual hubs and their interactions and comparison to the top degree-based hubs. To construct a network using CHAT, the user must provide a list of gene identifiers and associated numerical or categorical attributes in the text box in tab-delimited format, or upload the data as a csv or tab-delimited file via the upload button ( Figure 1) (.csv or .txt file types). The user can then specify which genes in the uploaded list are contextually important based on the user-provided contextual data (e.g. genes with > 2 fold-change in expression). The user then selects one of the databases in the PSICQUIC registry to query, and specifies the relevant species, ID type and interaction type for the query. The user can then choose to visualize the network using any of the layout algorithms available in Cytoscape. Clicking the OK button creates the network and a new tab in the results panel, which allows the user to visualize the network and to analyze the results further ( Figure 2). The results panel is split into several parts. In the first part, the parameters used to generate the network (database, species, id type and interaction type(s)) are displayed. The second panel allows the user to compare the top contextual hubs and the top degree-based hubs at the click of a button. By default, node size and node color are proportional to the node’s corrected p-value calculated by CHAT, such that the smaller the p-value (i.e. more statistically significant), the larger the node size and the darker the red coloring of the node. The user can customize the color scheme, however. In contrast, if the users selects “Show degree hubs”, the visualization changes and the node size and coloring will now be proportional to each node’s degree in the selected database. By default, CHAT displays the top 20 contextual hubs but the user can adjust this by using the slider provided. To investigate a single node in detail the user can employ CHAT’s “Node Analyzer” by right clicking on a node. This will limit the network view to show only the selected node and its interactors and will display a table at the bottom of the results panel tab with information on the node’s name, p-value and its interactors.

## Use case

Use case data 462 genes that have been reported to be up-regulated during Dengue fever infection
Copyright: © 2016 Muetze T et al. 2016 Data associated with the article are available under the terms of the Creative Commons Zero "No rights reserved" data waiver (CC0 1.0 Public domain dedication).

**Figure 3.**
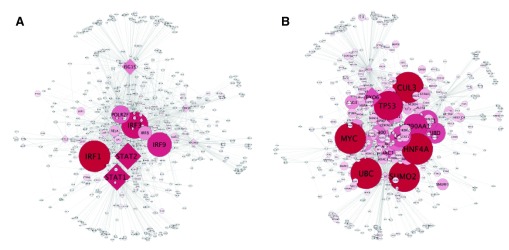
Visualization of a Dengue gene expression dataset. A CHAT network visualization comparing contextual hubs ( **A**) to degree-based hubs ( **B**) in a network constructed using InnateDB ^23, 24^.

As a demonstration of its potential utility and as validation, CHAT was used to construct a network using a dataset of 462 genes that have been reported to be up-regulated during Dengue fever, a mosquito-borne viral infection ^22^ (Ensembl gene IDs for these 462 genes are provided in Dataset 1). These 462 genes represent the contextual data for this case study. CHAT was used to construct a network of these genes and their first neighbor interactors using interaction data that was sourced from InnateDB ^23, 24^ via the PSICQUIC web service (InnateDB-All). A network of 4,910 nodes was generated. CHAT was then used to identify the top 20 conventional hub nodes (based solely on degree) and the top 20 contextual hub nodes in the network ( Figure 3). No nodes were in common in the two top 20 lists. InnateDB pathway analysis ^23, 24^ revealed that the top 20 degree-based hubs were enriched in pathways related to the cell cycle and cancer ( Supplementary Table 1), which is likely due to the fact that proteins involved in these processes tend to be highly connected in general. In comparison to degree-based hubs, the top 20 contextual hubs were statistically enriched in pathways related to the immune response to viral infection, such as the interferon signaling pathway; the Retinoic acid inducible gene-I (RIG-I) pathway; the Toll-like receptor (TLR) pathway; and the Janus kinase (JAK) - Signal Transducer and Activator of Transcription (STAT) pathway ( Supplementary Table 2). All of these pathways have been shown to play key roles in the host response to Dengue infection ^25, 26^. Indeed, many of the top 20 contextual hubs (but not degree-based hubs) were well-known transcription factors involved in the host interferon response including STAT1, STAT2 and the interferon regulatory factors (IRFs); IRF1, 3, 8 and 9, which is a key cellular response to viral infection including Dengue ^27, 28^. Another gene identified in the contextual hub analysis but not the degree-based analysis was interferon-stimulated gene 15 (ISG15). Cells in which ISG15 has been silenced have been shown to have significantly higher Dengue viral loads ^29^. The results of the pathway analysis were reinforced by a Gene Ontology analysis using innatedb.com ^23, 24^, which identified terms including cytokine-mediated signaling pathway, type I interferon signaling pathway, and innate immune response among the top 10 enriched terms (FDR < 0.05) for the contextual hubs but not the degree-based hubs ( Supplementary Table 3 and Supplementary Table 4).

## Conclusion

Through the integration of contextual information, such as gene or protein expression, contextual hub analysis as implemented in CHAT can identify context-specific hubs more relevant to the biological context under study, such as disease, treatment or cellular state. As shown in the above case study, these hubs are of more functional relevance than genes found through analysis based on degree only. Given the current emphasis on the importance of considering the network model of biological pathways and the ever-increasing abundance of high-throughput data, CHAT provides a valuable addition to the biologists’ computational toolkit in using a network-based approach to help prioritize genes of interest for further investigation or drug discovery. In the future, CHAT can be extended to include the contextual analysis of other network features such as network bottlenecks.

## Data availability

The data referenced by this article are under copyright with the following copyright statement: Copyright: © 2016 Muetze T et al.

Data associated with the article are available under the terms of the Creative Commons Zero "No rights reserved" data waiver (CC0 1.0 Public domain dedication). 

F1000Research: Dataset 1. Use case data: 462 genes that have been reported to be up-regulated during Dengue fever infection, 10.5256/f1000research.9118.d128126 ^30^

## Software availability

Software available from: http://apps.cytoscape.org/apps/chat

Latest source code: https://bitbucket.org/dynetteam/chat

Archived source code at time of publication: http://www.dx.doi.org/10.5281/zenodo.56496 ^31^

Manual/Tutorial: https://bitbucket.org/dynetteam/chat/downloads

License: Lesser GNU Public License 3.0
