# Supplementary material for: Contextual Hub Analysis Tool (CHAT): A Cytoscape app for identifying contextually relevant hubs in biological networks
Source: F1000Res. 2016 Aug 30;5:1745. Originally published 2016 Jul 19. [Version 2] doi: 10.12688/f1000research.9118.2 (PMC5105880; doi:10.12688/f1000research.9118.2)
Supplement: Peer review history

# Peer review: Referee response for version 2

**DOI:** 10.5256/f1000research.10240.r16856

**Published:** 2016-11-01

**Authors:** Christopher K. Tuggle, Haibo Liu

**Affiliations:** 1 Bioinformatics and Computational Biology Program, Iowa State University, Ames, IA, USA

This Cytoscape app “CHAT” is valuable given its improvement over conventional biological network analysis methods by considering the context of network analysis. It is a good addition to the Cytoscape toolkits. However, we suggest some modifications that might make the paper more readable and the method more applicable.

 Suggested Modifications to text

  

1. In the “Operation” section, “The identification of the top contextual hubs consists of three primary steps: 1) input of a user-supplied gene list and contextual data, 2) network construction and statistical analysis to identify nodes that preferentially interact with contextual nodes and 3) visualization of the top contextual hubs and their interactions and comparison to the top degree-based hubs.” should include four steps to be consistent with the following statements: 1) input a gene list, **2) database selection**, 3) network construction and statistical analysis, 4) visualization of the top conceptual hubs and their interactions and comparison to the top degree-based hubs.
2. The “implementation” and “Operation” sections in the Methods are somewhat redundant. For example, “The OK button triggers Cytoscape’s TaskManager to run a task that initiates the network construction and adds a tab to the results panel that provides functionality to further modify and analyze the network” and “A right click on a node brings up an option to activate the “Node Analyzer”mode”. It will be more appropriate if both these sentences are moved to the “Operation” section. The “Implementation” section should focus on the description of functionalities provide by CHAT and the application interfaces (APIs) are implemented, while how to conduct network analysis should be in the “Operation” part. 

Suggested Modifications to future versions of CHAT

  

1. One question is the background selection criteria. As the authors mentioned, this step is very important and directly affects the later statistical test results. An alternative/better background might be the set of genes/proteins expressed under the condition used to create the original dataset, such as genes expressed in given the cell type, tissue, or treatment. This might eliminate the irrelevant nodes and reduce the number of tests needed.
2. In the current CHAT version, only the first-order neighbor-interactors are allowed to be considered which is generally most important and might be enough in most situations. But when the resulting network is small, the user might not be able to perform further analysis. So if the option of higher-order interactors is provided, the tool will be more versatile.
3. The authors mentioned that only databases in the PSICQUIC registry with at least 10,000 interactions are included. This is kind of arbitrary. While it is appreciated that other DB available in Cytoscape can be used with this tool, the usefulness of the databases should be determined case by case. So we suggest the author provide the user all the available choices of interaction databases.
4. We suggest a different method might be more appropriate for multiple testing correction.

The authors state that “a method widely used in bioinformatics to avoid high false discovery rates. The Bonferroni approach is widely considered to be too strict.” This could be reworded as “a method widely used in bioinformatics to avoid high false discovery rates, instead of the Bonferroni approach which is widely considered to be too strict.”

 However, the author assumed the genes in the network are independent by using the “BH” method. To be more realistic, we suggest the “BY” method for correction of the multiple testing. See Benjamini and Yekutieli: The control of the false discovery rate in multiple testing under dependency *Ann. Statist. *2001; **29** (1165-1188)

We have read this submission. We believe that we have an appropriate level of expertise to confirm that it is of an acceptable scientific standard.

# Peer review: Referee response for version 1

**DOI:** 10.5256/f1000research.9812.r15065

**Published:** 2016-08-09

**Authors:** Sandra Orchard, Pablo Porras Millán

**Affiliations:** 1 Wellcome Trust Genome Campus, European Molecular Biology Laboratory-European Bioinformatics Institute, Hinxton, UK

This is a well written technical paper, clearly outlining a new Cytoscape App in terms that would make it easy for a new user, with some familiarity with Cytoscape, to download, install and use. The ability to generate contextual hubs is currently not possible with existing Cytoscape Apps, so this is a valuable addition to the collection. A couple of queries and some minor points for correction:

1. The application searches for first-neighbour interactions of molecules in the list presented to it. It did not appear to search for interaction between members of the list, which should not affect the contextual nodes selection much, but will alter the degree-based hubs. This should be commented on, or the documentation made clearer if we are incorrect with this observation. To bypass this problem and make the user more aware of this limitation, the tool should be able to provide more control over how the network is constructed, for example providing the option to exclude first neighbours.
2. Can this application be made to work with an existing network?
3. The text is entirely gene-centric and may leave an inexperienced use under the impression is is only usable for gene-expression data whereas it is equally useful for the analysis of proteomic data and works with UniProtKB identifiers. Whilst I realise this is apparent to anyone who downloads the app, it may well be worth adding a sentence to both the Summary or Introduction of this paper, and also the description in the App store just to make this very clear to naive users.
4. It may also be worth adding the reference to the 2013 PSICQUIC paper as well as the original as I personally find it more informative and again, may be helpful to the inexperienced user.

We have read this submission. We believe that we have an appropriate level of expertise to confirm that it is of an acceptable scientific standard.

## References

[1] del-ToroNDumousseauMOrchardSJimenezRCGaleotaELaunayGGollJBreuerKOnoKSalwinskiLHermjakobH: A new reference implementation of the PSICQUIC web service. *Nucleic Acids Res*.2013;41(Web Server issue) : 10.1093/nar/gkt392 W601-6 10.1093/nar/gkt392 23671334PMC3977660

# Response

**Published:** 2016-08-22

**Authors:** Tanja Muetze

**Affiliations:** SAHMRI, Australia

Thank you very much for your thoughtful review. Below we have addressed each of the points raised.

***The application searches for first-neighbour interactions of molecules in the list presented to it. It did not appear to search for interaction between members of the list, which should not affect the contextual nodes selection much, but will alter the degree-based hubs. This should be commented on, or the documentation made clearer if we are incorrect with this observation. To bypass this problem and make the user more aware of this limitation, the tool should be able to provide more control over how the network is constructed, for example providing the option to exclude first neighbours.***

 This observation is incorrect. CHAT does include interactions between members of the uploaded list. Perhaps what you mean is that interactions between the first neighbors of the uploaded list of genes/proteins are not considered? These are actually also considered in CHAT’s calculations – they are just excluded from the visualization to avoid unhelpful "hairball" visualizations. We have now clarified this point in the paper. We are not sure why a user would want to exclude first neighbors – this information is needed in CHAT to identify the contextual hubs.

***Can this application be made to work with an existing network?***

 We agree that this would be a very nice feature, unfortunately it is actually very difficult to do with the current design of CHAT. CHAT identifies hub nodes that interact with more "contextual" nodes than statistically expected using a hypergeometric test. This test is reliant on calculating, *N,* the number of genes with at least one interaction in the database queried to estimate the background expectation. This parameter would not be known or easily estimated in a user-supplied network as CHAT wouldn't know what database or the data in the database when the network was constructed by the user. One of the nice features of CHAT is that the network is constructed using the latest data available via a PSICQUIC query.

***The text is entirely gene-centric and may leave an inexperienced use under the impression is is only usable for gene-expression data whereas it is equally useful for the analysis of proteomic data and works with UniProtKB identifiers. Whilst I realise this is apparent to anyone who downloads the app, it may well be worth adding a sentence to both the Summary or Introduction of this paper, and also the description in the App store just to make this very clear to naive users.***

 Good point. We have now edited the text to clarify that protein as well as gene ids can be used to construct the network in CHAT.

***It may also be worth adding the reference to the 2013 PSICQUIC paper as well as the original as I personally find it more informative and again, may be helpful to the inexperienced user.***

 We have now added the suggested reference to the paper.

 Again, we thank you for comments and suggestions.
